# Joint Resource Allocation in Secure OFDM Two-Way Untrusted Relay System

**DOI:** 10.3390/s22062398

**Published:** 2022-03-21

**Authors:** Yifeng Jin, Xunan Li, Guocheng Lv, Meihui Zhao, Ye Jin

**Affiliations:** 1 School of Electronics, Peking University, Beijing 100871, China; jinyifeng@pku.edu.cn (Y.J.); meihui_zhao@pku.edu.cn (M.Z.); jinye@pku.edu.cn (Y.J.); 2 National Computer Network Emergency Response Technical Team Coordination Center of China, Beijing 100029, China; lixunan@cert.org.cn

**Keywords:** physical layer security, OFDM two-way system, untrusted relay, joint resource allocation

## Abstract

The security issue of wireless communication is a common concern because of its broadcast nature, especially when the relay becomes an eavesdropper. In the orthogonal frequency division multiplexing (OFDM) relay system, when the relay is untrusted, the security of the system faces serious threats. Although there exist some resource allocation schemes in a single-carrier system with untrusted relaying, it is difficult to apply them to the multi-carrier system. Hence, a resource allocation scheme for the multi-carrier system is needed. Compared to the one-way relay system, a two-way relay system can improve the data transmission efficiency. In this paper, we consider joint secure resource allocation for a two-way cooperative OFDM system with an untrusted relay. The joint resource allocation problem of power allocation and subcarrier pairing is formulated to maximize the sum secrecy rate of the system under individual power constraints. To solve the non-convex problem efficiently, we propose an algorithm based on the alternative optimization method. The proposed algorithm is evaluated by simulation results and compared with the benchmarks in the literature. According to the numerical results, in a high signal-to-noise ratio (SNR) scenario, the proposed algorithm improves the achievable sum secrecy rate of the system by more than 15% over conventional algorithms.

## 1. Introduction

Due to the broadcast nature of wireless communication, the threats of eavesdropping and information leakages have increased sharply and the security issue becomes a common concern. The traditional encryption technology uses cryptographic methods in the upper layer of the system, which is crackable and insufficient with the rapid growth of computing power. Therefore, secure communication in the lower layer of the system has been studied and the physical layer security has become a frontier research. Unlike cryptographic methods, the principle of physical layer security technology is to use the difference between the legal channel and the eavesdropping channel to achieve secure communication of the system, which is also its advantage [1,2]. The concept of physical layer security is first introduced by Wyner in a one-hop communication system [3]. With the wide application of relays, many researchers have extended this technology to relay systems [4,5,6,7]. In order to utilize the limited resource of the communication system to maximize the secrecy rate, efficient resource allocation is essential in physical layer security.

The two-way relay communication systems has attracted much attention in the past few years due to its ability in combating the half-duplex constraint of relay nodes and improving the data transmission efficiency [8,9,10]. Due to the advantages of physical layer security, many studies on two-way cooperative communication systems have considered security issue from a physical layer perspective. The research scenarios can be divided into two cases: (1) External eavesdropper [11,12], in which an illegitimate eavesdropper that does not belong to the network tries to decode the confidential information, and (2) untrusted relay [13,14,15,16,17,18,19], in which the relay is assumed to be untrustworthy and acts as an eavesdropper.

In the two-way untrusted relay system, the physical security was first studied in [13], in which the authors proved this system can achieve secure communication without external friendly jammer. The authors in [14] employed artificial noise and studied power allocation in the two-way untrusted relay system with channel estimation errors. Ref.  [15] studied the power allocation in the two-way system with multiple untrusted relays. In the two-way untrusted relay system with multi-antenna, the authors in [16] proposed a joint beamforming and suboptimal power allocation scheme to maximize the sum secrecy rate of the system. The problem of optimal power allocation for two-way untrusted relaying networks with an external jammer was examined in [17]. In [18], the authors studied secure relay selection for two-way untrusted relaying networks. Secure beamforming for full-duplex multiple-input–multiple-output (MIMO) two-way untrusted relay systems was considered in [19]. In other scenarios, the authors in [20] proposed machine learning techniques to conserve the position confidentiality of roaming position-based services (PBSs) users. In [21], the authors proposed a whale optimization algorithm to solve the resource allocation problem in an Internet of Things (IoT) system to reduce the total communication cost.

The above related works are all based on the single-carrier system, so the resources that need to be allocated in the system are the transmission power only. In two-way relay assisted orthogonal frequency division multiplexing (OFDM) systems, channel gains of one subcarrier in one hop differ from another hop, and system capacity can be maximized by subcarrier pairing and power allocation [22]. The joint resource allocation in the secure OFDM one-way system was studied in some previous works [23,24,25]; however, due to the differences in system models, the existing resource allocation schemes cannot be applied in an OFDM two-way untrusted relay system. A summary of related work is presented in Table 1. To the best of the authors’ knowledge, secure resource allocation jointly considering subcarrier pairing and power allocation for a two-way cooperative OFDM system with untrusted relaying has not been studied in the literature.

In this paper, our main innovation is to study the secure resource allocation jointly considering subcarrier pairing and power allocation for a two-way cooperative OFDM system with untrusted relaying, including constructing the system model, formulating the optimization problem, and proposing an effective algorithm to solve the non-convex problem. Our goal is to achieve the secure communication in the OFDM two-way communication system with untrusted relaying. We use resource allocation to enhance the security performance of the system by maximizing the sum secrecy rate of the system under individual power constraints of each transmit node. The key idea of our resource allocation algorithm is to decouple the non-convex optimization problem into several brief subproblems and relax them to convex ones.

The major contribution of this paper are twofold:

1. We introduce a two-way cooperative OFDM system with an untrusted relay and formulate the resource allocation problem to maximize the sum secrecy rate under individual power constraints, which include the subcarrier pairing and power allocation.

2. To solve the problem, we propose a joint resource allocation algorithm based on alternative optimization method. The problem is solved by divided into four subproblems. In particular, we show that the complexity of the solution is polynomial in the number of subcarriers. According to the numerical results, in a high signal-to-noise ratio (SNR) scenario, the proposed algorithm improves the achievable sum secrecy rate of the system by more than 15% over conventional algorithms.

The remainder of this paper is organized as follows. Section 2 describes the system model and derives the formulation of the sum secrecy rate. Section 3 formulates the optimization problem and presents an efficient joint resource allocation algorithm based on alternative optimization (AO). Section 4 demonstrates the simulation results to illustrate the performance of the proposed algorithm. Finally, this paper is concluded in Section 5.

## 2. System Model

**Figure 1:**
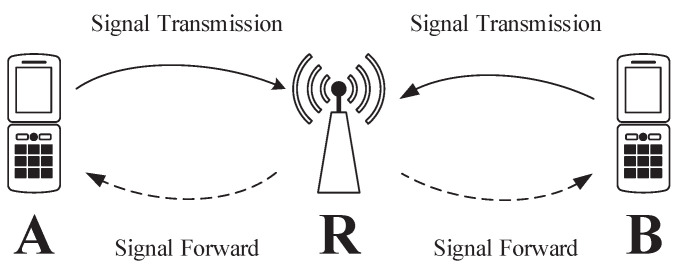
An OFDM two-way system with an untrusted relay. Solid lines: signal transmission on subcarriers in the first slot. Dashed lines: signal forward transmission on subcarriers in the second slot.

We consider a two-way cooperative OFDM system with untrusted relaying, as shown in Figure 1, where two users, denoted as A and B, wish to exchange confidential information via an untrusted relay R. The two-way relay operates in a half-duplex mode using the amplify-and-forward (AF) protocol. All communication nodes are assumed to be equipped with a single antenna. We assume that there are no direct links between A and B due to the long distance between them. The users-to-relay channels are considered to be reciprocal, occupying the same bandwidth and experiencing frequency-selective fading. Each OFDM channel is composed of *N* orthogonal subcarriers.

Particularly, the relay is assumed trusted at the service level and untrusted at the data level, as in [26]. Service-level trust entails following the AF protocol as expected. This involves for relays to feedback true CSI, adapt their power according to system schedule, and forward the amplified version of received signal without modification. Since the relay is data-level untrusted, the source imposes security constraints on relays. This is to prevent the untrusted relay from extracting useful information from its received signal.

The transmission from the users to the relay is on a timeframe basis with each frame consisting of multiple OFDM symbols. Each frame is further divided into two time slots. In the first time slot, both A and B send signals to R simultaneously on all subcarriers. We denote the channel coefficients of A to R and B to R on the *i*-th subcarrier as $h_{i,A}$ and $h_{i,B}$, respectively, for i∈{1,...,N}. We further assume that the transmit powers of A and B on the *i*-th subcarrier are $P_{i,A}$ and $P_{i,B}$, respectively. Then, the received signal at R on the *i*-th subcarrier in the first time slot can be given by

(1) $$\begin{array}{c} y_{i,R}=\sqrt{P_{i,A}}h_{i,A}w_{i,A}+\sqrt{P_{i,B}}h_{i,B}w_{i,B}+n_{i,R}, \end{array}$$

where $w_{i,A}$ and $w_{i,B}$ denote symbol of A and B’s signal on the *i*-th subcarrier, respectively. $n_{i,R}$ denotes the additive white Gaussian noise (AWGN) signal at the relay on the *i*-th subcarrier and the noise power is $\sigma_{n}^{2}$.

In the second time slot, R amplifies the received signals $y_{i,R}$ on the *i*-th subcarrier with a constant gain and forwards them to both A and B on the $i^{\prime }$-th subcarrier. Note that the subcarrier index $i^{\prime }$ may not be as same as *i* and they form a subcarrier pair $\left(i,i^{\prime }\right)$, and each subcarrier in A to R is paired with only one subcarrier in B to R. Since each subcarrier has different channel gains, subcarrier pairing can utilize subcarrier diversity to enhance system performance. We denote the transmit power of R on the $i^{\prime }$-th subcarrier as $P_{i^{\prime },R}$. Let $\beta_{i,i^{\prime }}$ represent the power gain at R to normalize the power of the signal transmitted, i.e.,

(2) $$\begin{array}{c} \beta_{i,i^{\prime }}=\frac{P_{i^{\prime },R}}{|h_{i,A}{|}^{2}P_{i,A}+{\left|h_{i,B}\right|}^{2}P_{i,B}+\sigma_{R}^{2}}. \end{array}$$

Following that, the received signal at A and B from R on the $i^{\prime }$-th subcarrier in the second time slot can be given by

(3) $$\begin{array}{c} y_{i^{\prime },A}=\sqrt{\beta_{i,i^{\prime }}}\sqrt{P_{i,A}}h_{i^{\prime },A}h_{i,A}w_{i,A}+\sqrt{\beta_{i,i^{\prime }}}\sqrt{P_{i,B}}h_{i^{\prime },A}h_{i,B}w_{i,B}+\sqrt{\beta_{i,i^{\prime }}}h_{i^{\prime },A}n_{i,R}+n_{i^{\prime },A}, \end{array}$$

and

(4) $$\begin{array}{c} y_{i^{\prime },B}=\sqrt{\beta_{i,i^{\prime }}}\sqrt{P_{i,A}}h_{i^{\prime },B}h_{i,A}w_{i,A}+\sqrt{\beta_{i,i^{\prime }}}\sqrt{P_{i,B}}h_{i^{\prime },B}h_{i,B}w_{i,B}+\sqrt{\beta_{i,i^{\prime }}}h_{i^{\prime },B}n_{i,R}+n_{i^{\prime },B}, \end{array}$$

where $n_{i^{\prime },A}$ and $n_{i^{\prime },B}$ denote the AWGN at A and B on the $i^{\prime }$-th subcarrier with variances $\sigma_{n}^{2}$. Assuming that both user A and B can perfectly estimate the channel state information (CSI) for channels $h_{i^{\prime },A}$ and $h_{i,B}$, the self-interference terms can be eliminated perfectly at both users (first term in (3) and second term in (4)). Therefore, the received signal at A and B can be expressed as

(5) $$\begin{array}{cc} y_{i^{\prime },A} & =\sqrt{\beta_{i,i^{\prime }}}\sqrt{P_{i,B}}h_{i^{\prime },A}h_{i,B}w_{i,B}+\sqrt{\beta_{i,i^{\prime }}}h_{i^{\prime },A}n_{i,R}+n_{i^{\prime },A}, \end{array}$$

(6) $$\begin{array}{cc} y_{i^{\prime },B} & =\sqrt{\beta_{i,i^{\prime }}}\sqrt{P_{i,A}}h_{i^{\prime },B}h_{i,A}w_{i,A}+\sqrt{\beta_{i,i^{\prime }}}h_{i^{\prime },B}n_{i,R}+n_{i^{\prime },B}. \end{array}$$

Then, the resultant SNR at A and B on the subcarrier pair $\left(i,i^{\prime }\right)$ can be represented as

(7) $$\begin{array}{c} \gamma_{i,i^{\prime },A}=\frac{\beta_{i,i^{\prime }}P_{i,B}|h_{i^{\prime },A}{|}^{2}{\left|h_{i,B}\right|}^{2}}{\beta_{i,i^{\prime }}{\left|h_{i^{\prime },A}\right|}^{2}\sigma_{n}^{2}+\sigma_{n}^{2}}=\frac{\alpha_{i,B}\alpha_{i^{\prime },A}P_{i,B}P_{i^{\prime },R}}{\alpha_{i,A}P_{i,A}+\alpha_{i,B}P_{i,B}+\alpha_{i^{\prime },A}P_{i^{\prime },R}+1}, \end{array}$$

and

(8) $$\begin{array}{c} \gamma_{i,i^{\prime },B}=\frac{\beta_{i,i^{\prime }}P_{i,A}|h_{i^{\prime },B}{|}^{2}{\left|h_{i,A}\right|}^{2}}{\beta_{i,i^{\prime }}{\left|h_{i^{\prime },B}\right|}^{2}\sigma_{n}^{2}+\sigma_{n}^{2}}=\frac{\alpha_{i,A}\alpha_{i^{\prime },B}P_{i,A}P_{i^{\prime },R}}{\alpha_{i,A}P_{i,A}+\alpha_{i,B}P_{i,B}+\alpha_{i^{\prime },B}P_{i^{\prime },R}+1}, \end{array}$$

where $\alpha_{i,A}={\left|h_{i,A}\right|}^{2}/\sigma_{n}^{2}$ and $\alpha_{i,B}={\left|h_{i,B}\right|}^{2}/\sigma_{n}^{2}$ are effective channel coefficients. Then, the transmission rate at A and B on the subcarrier pair $\left(i,i^{\prime }\right)$ can be expressed as $R_{i,i^{\prime },A}=\frac{1}{2}\log_{2}\left(1+\gamma_{i,i^{\prime },A}\right)$ and $R_{i,i^{\prime },B}=\frac{1}{2}\log_{2}\left(1+\gamma_{i,i^{\prime },B}\right)$, respectively.

As mentioned before, the relay is considered to be untrusted and tries to eavesdrop on the confidential signal. The untrusted relay employs successive the interference cancellation (SIC) method to decode the mixed signal, which requires the relay to decode one user’s signal and remove it from the mixed signals before decoding another user’s signal. This creates two situations that we need to discuss separately: (1) The SIC is successful, which means the relay successfully decodes one user’s signal. In this case, the relay will not experience interference while it decodes another user’s signal. Since the signal will weaken in the transmission of relay forward to the user, the eavesdropping rate of relay must be greater than the transmission rate of the user, so the system cannot achieve secure communication and the resource allocation is meaningless. (2) The SIC is unsuccessful, then the untrusted relay decodes one of the users’ signals by treating the other user as noise, which is called the single-user decode mode [27]. In this case, the resource allocation can achieve secure communication of the system. Therefore, in the following derivation, the untrusted relay adopts the single-user decode mode. The eavesdropping rate at R on A and B over the *i*-th subcarrier can be computed from (1) and is given by

(9) $$\begin{array}{c} R_{i,\mathrm{RA}}=\frac{1}{2}\log_{2}\left(1+\frac{P_{i,B}{\left|h_{i,B}\right|}^{2}}{P_{i,A}{\left|h_{i,A}\right|}^{2}+\sigma_{n}^{2}}\right)=\frac{1}{2}\log_{2}\left(1+\frac{\alpha_{i,B}P_{i,B}}{\alpha_{i,A}P_{i,A}+1}\right), \end{array}$$

and

(10) $$\begin{array}{c} R_{i,\mathrm{RB}}=\frac{1}{2}\log_{2}\left(1+\frac{P_{i,A}{\left|h_{i,A}\right|}^{2}}{P_{i,B}{\left|h_{i,B}\right|}^{2}+\sigma_{n}^{2}}\right)=\frac{1}{2}\log_{2}\left(1+\frac{\alpha_{i,A}P_{i,A}}{\alpha_{i,B}P_{i,B}+1}\right). \end{array}$$

Therefore, the sum secrecy rate of the system on subcarrier pair $\left(i,i^{\prime }\right)$ can be formulated as

(11) $$\begin{array}{c} R_{i,i^{\prime }}^{S}={\left(R_{i,i^{\prime },A}-R_{i,\mathrm{RA}}\right)}^{+}+{\left(R_{i,i^{\prime },B}-R_{i,\mathrm{RB}}\right)}^{+}, \end{array}$$

where ${\left(x\right)}^{+}=\max\left(x,0\right)$.

In this paper, our aim is to jointly design the resource allocation scheme of power allocation and subcarrier pairing to maximize the sum secrecy rate of the system, with the constraints of individual power budgets per node. We define $P=\{P_{i,A},P_{i,B},P_{i^{\prime },R}\}$ as the set of power allocation scheme, and it satisfies the individual power constraints, which are

(12) $$\begin{array}{cc} \; & \sum_{i=1}^{N}P_{i,A}\leq P_{A}, \end{array}$$

(13) $$\begin{array}{cc} \; & \sum_{i=1}^{N}P_{i,B}\leq P_{B}, \end{array}$$

(14) $$\begin{array}{cc} \; & \sum_{i^{\prime }=1}^{N}P_{i^{\prime },R}\leq P_{R}, \end{array}$$

where $P_{A}$, $P_{B}$, and $P_{R}$ denote the transmitting power budgets on the user *A*, the user *B*, and the relay *R*, respectively.

In addition to the power constraints, the system must also satisfy the subcarrier pairing constraint that guarantees that each subcarrier is paired strictly with one subcarrier. We define $\rho =\{\rho_{i,i^{\prime }}\}$ as the set of subcarrier pairing scheme; $\rho_{i,i^{\prime }}=1$ indicates that the *i*-th subcarrier in the first slot is paired with the $i^{\prime }$-th subcarrier in the second slot. The subcarrier pairing constraint can be given by

(15) $$\begin{array}{c} \sum_{i^{\prime }=1}^{N}\rho_{i,i^{\prime }}=1,\;\forall i,\;\;\;\;\;\sum_{i=1}^{N}\rho_{i,i^{\prime }}=1,\;\forall i^{\prime }, \end{array}$$

## 3. Resource Allocation for Sum Secrecy Rate Maximization

The optimization problem can be formulated as

(16) $$\begin{array}{cc} \; & \max_{\left\{P,\rho \right\}}\sum_{i=1}^{N}\sum_{i^{\prime }=1}^{N}\rho_{i,i^{\prime }}R_{i,i^{\prime }}^{S}\\ s.t.\; & \sum_{i=1}^{N}P_{i,A}\leq P_{A},\sum_{i=1}^{N}P_{i,B}\leq P_{B},\sum_{i^{\prime }=1}^{N}P_{i^{\prime },R}\leq P_{R},\\ \; & \sum_{i^{\prime }=1}^{N}\rho_{i,i^{\prime }}=1,\;\forall i,\sum_{i=1}^{N}\rho_{i,i^{\prime }}=1,\;\forall i^{\prime }. \end{array}$$

The optimization problem in (16) is a non-convex mixed-integer programming, which is NP-hard. Since there exist four optimization variables, i.e., $P_{i,A}$, $P_{i,B}$, $P_{i^{\prime },R}$, and $\rho_{i,i^{\prime }}$ in (16), we can decompose the primal problem into four subproblems by using the alternating optimization (AO) method, which is widely used in research related to resource allocation [28,29,30]. In the AO method, the optimal resource allocation scheme of the optimization problem is obtained by solving the subproblems in sequence, which are discussed in the following subsections.

### 3.1. Power Allocation Scheme for User A

Assuming that the other optimization variables, i.e., $P_{i,B}$, $P_{i^{\prime },R}$, and $\rho_{i,i^{\prime }}$, are given, and we define the $i^{\prime }$ as the given subcarrier index in the second time slot paired with the *i*-th subcarrier in the first time slot, then the subproblem of power allocation for user A can be given as

(17) $$\begin{array}{cc} \; & \max_{\left\{P_{i,A}\right\}}\sum_{i=1}^{N}R_{i,i^{\prime }}^{S}\\ \; & s.t.\;\sum_{i=1}^{N}P_{i,A}\leq P_{A}, \end{array}$$

which is still a non-convex problem. Since most of the impact caused by the power allocation for the user A is reflected in the transmission rate from the user A to the user B and the eavesdropping rate at R on B, we can approximate the optimization problem in (17) as

(18) $$\begin{array}{cc} \; & \max_{\left\{P_{i,A}\right\}}\sum_{i=1}^{N}\left(R_{i,i^{\prime },B}-R_{i,\mathrm{RB}}\right).\\ \; & s.t.\;\sum_{i=1}^{N}P_{i,A}\leq P_{A}. \end{array}$$

**Proposition** **1.** *The optimization problem in (18) is convex.*

**Proof.**  See Appendix A.    □

Therefore, we can solve the convex problem in (18) by dual method [31]. We denote $\lambda_{A}\geq 0$ as the dual variable associated with the power constraints in the user A. The dual function can be defined as

(19) $$\begin{array}{c} g\left(\lambda_{A}\right)=\max_{P_{i,A}}L_{A}\left(P_{i,A},\lambda_{A}\right), \end{array}$$

where the Lagrangian is

(20) $$\begin{array}{c} L_{A}\left(P_{i,A},\lambda_{A}\right)=\sum_{i=1}^{N}\frac{1}{2}\log_{2}\left(\frac{a_{A}P_{i,A}+b_{A}}{P_{i,A}^{2}+c_{A}P_{i,A}+b_{A}}\right)+\lambda_{A}\left(P_{A}-\sum_{i=1}^{N}P_{i,A}\right), \end{array}$$

where $a_{A},b_{A}$ and $c_{A}$ are coefficients determined by the channel gains as  

$$\begin{array}{cc} a_{A} & =\left(\alpha_{i^{\prime },B}P_{i^{\prime },R}+1\right)\left(\alpha_{i,B}P_{i,B}+1\right)/\alpha_{i,A},\\ b_{A} & =\left(\alpha_{i^{\prime },B}P_{i^{\prime },R}+\alpha_{i,B}P_{i,B}+1\right)\left(\alpha_{i,B}P_{i,B}+1\right)/\alpha_{i,A}^{2},\\ c_{A} & =\left(\alpha_{i^{\prime },B}P_{i^{\prime },R}+2\alpha_{i,B}P_{i,B}+2\right)/\alpha_{i,A}. \end{array}$$

Computing the dual function $g(\lambda_{A})$ requires us to determine the optimal $P_{i,A}$ at the given dual variable $\lambda_{A}$. In the following we present the derivations in detail.

#### 3.1.1. Optimizing the Primal Variables $P_{i,A}$ for Given $\lambda_{A}$

By applying Karush–Kuhn–Tucker (KKT) conditions [32], we can obtain the optimal power allocation scheme $P_{i,A}^{*}\left(\lambda_{A}\right)$. Specifically, $P_{i,A}^{*}\left(\lambda_{A}\right)$ is the non-negative real root of the following cubic equation:

(21) $$\begin{array}{c} A_{A}P_{i,A}^{3}+B_{A}P_{i,A}^{2}+C_{A}P_{i,A}+D_{A}=0, \end{array}$$

where $A_{A},B_{A},C_{A}$, and $D_{A}$ are coefficients determined by the channel gains and the dual variable $\lambda_{A}$ as

$$\begin{array}{cc} A_{A} & =a_{A},\\ B_{A} & =b_{A}+a_{A}c_{A}+a_{A}/\left(2\ln2\lambda_{A}\right),\\ C_{A} & =b_{A}\left(a_{A}+c_{A}\right)+b_{A}/\left(2\ln2\lambda_{A}\right),\\ D_{A} & =b_{A}^{2}+b_{A}\left(c_{A}-a_{A}\right)\left(2\ln2\lambda_{A}\right). \end{array}$$

Then the dual function can be further written as

(22) $$\begin{array}{c} g\left(\lambda_{A}\right)=\max_{P_{i,A}^{*}}L_{A}\left(P_{i,A}^{*},\lambda_{A}\right), \end{array}$$

#### 3.1.2. Optimizing the Dual Variable $\lambda_{A}$

After computing $g(\lambda_{A})$, we now solve the standard dual optimization problem which is

(23) $$\begin{array}{cc} \; & \min_{\lambda_{A}}g\left(\lambda_{A}\right)\\ \; & s.t.\;\lambda_{A}\geq 0. \end{array}$$

Since the dual function is always convex [32], the dual optimization problem in (23) can be solved by subgradient-based methods with global convergence. The subgradient of $g(\lambda_{A})$ can be derived as

(24) $$\begin{array}{cc} \; & △\lambda_{A}=P_{A}-\sum_{i=1}^{N}P_{i,A}^{*}\left(\lambda_{A}\right). \end{array}$$

The dual variable can be updated as $\lambda_{A}^{\left(l+1\right)}=\lambda_{A}^{\left(l\right)}+\epsilon^{\left(l\right)}△\lambda_{A}$, where *l* is the number of iterations and $\epsilon^{\left(l\right)}$ is the diminishing update step size to guarantee the convergence of the subgradient method.

### 3.2. Power Allocation Scheme for User B

Keeping the given and obtained variables, i.e., $P_{i,A}^{*}$, $P_{i^{\prime },R}$, and $\rho_{i,i^{\prime }}$, the subproblem of power allocation for user B can be given as

(25) $$\begin{array}{cc} \; & \max_{\left\{P_{i,B}\right\}}\sum_{i=1}^{N}R_{i,i^{\prime }}^{S}\\ \; & s.t.\;\sum_{i=1}^{N}P_{i,B}\leq P_{B}, \end{array}$$

which is still a non-convex problem. Since most of the impact caused by the power allocation for the user B is reflected in the transmission rate from the user B to the user A and the eavesdropping rate at R on A, we can approximate the optimization problem in (25) as

(26) $$\begin{array}{cc} \; & \max_{\left\{P_{i,B}\right\}}\sum_{i=1}^{N}\left(R_{i,i^{\prime },A}-R_{i,\mathrm{RA}}\right),\\ \; & s.t.\;\sum_{i=1}^{N}P_{i,B}\leq P_{B}. \end{array}$$

**Proposition** **2.** *The optimization problem in (26) is convex.*

**Proof.**  See Appendix B.    □

Therefore, the subproblem in (26) can be similarly solved as problem in (18) by the dual method. We denote $\lambda_{B}\geq 0$ as the dual variable associated with the power constraints in the user B. After similar derivations, $P_{i,B}^{*}\left(\lambda_{B}\right)$ is the non-negative real root of the following cubic equation:

(27) $$\begin{array}{c} A_{B}P_{i,B}^{3}+B_{B}P_{i,B}^{2}+C_{B}P_{i,B}+D_{B}=0, \end{array}$$

where $A_{B},B_{B},C_{B}$, and $D_{B}$ are coefficients determined by the channel gains and the dual variable $\lambda_{B}$ as

$$\begin{array}{cc} A_{B} & =a_{B},\\ B_{B} & =b_{B}+a_{B}c_{B}+a_{B}/\left(2\ln2\lambda_{B}\right),\\ C_{B} & =b_{B}\left(a_{B}+c_{B}\right)+b_{B}/\left(2\ln2\lambda_{B}\right),\\ D_{B} & =b_{B}^{2}+b_{B}\left(c_{B}-a_{B}\right)\left(2\ln2\lambda_{B}\right), \end{array}$$

where $a_{B},b_{B}$ and $c_{B}$ are coefficients determined by the channel gains as

$$\begin{array}{cc} a_{B} & =\left(\alpha_{i^{\prime },A}P_{i^{\prime },R}+1\right)\left(\alpha_{i,A}P_{i,A}+1\right)/\alpha_{i,B},\\ b_{B} & =\left(\alpha_{i^{\prime },A}P_{i^{\prime },R}+\alpha_{i,A}P_{i,A}+1\right)\cdot \left(\alpha_{i,A}P_{i,A}+1\right)/\alpha_{i,B}^{2},\\ c_{B} & =\left(\alpha_{i^{\prime },A}P_{i^{\prime },R}+2\alpha_{i,A}P_{i,A}+2\right)/\alpha_{i,B}. \end{array}$$

The dual variable $\lambda_{B}$ can be obtained by subgradient-based methods with global convergence as the solution of the problem in (23).

### 3.3. Power Allocation Scheme for Relay R

Keeping the given and obtained variables, i.e., $P_{i,A}^{*}$, $P_{i,B}^{*}$, and $\rho_{i,i^{\prime }}$, the subproblem of power allocation for relay R can be given as

(28) $$\begin{array}{cc} \; & \max_{\left\{P_{i^{\prime },R}\right\}}\sum_{i=1}^{N}R_{i,i^{\prime }}^{S}\\ \; & s.t.\;\sum_{i^{\prime }=1}^{N}P_{i^{\prime },R}\leq P_{R}. \end{array}$$

**Proposition** **3.** *The optimization problem in (28) is convex.*

**Proof.**  See Appendix C.    □

Therefore, the subproblem in (28) can be similarly solved as the problem in (18) by the dual method. We denote $\lambda_{R}\geq 0$ as the dual variable associated with the power constraints in the relay R. After similar derivations, $P_{i^{\prime },R}^{*}\left(\lambda_{R}\right)$ is the non-negative real root of the following quartic equation:

(29) $$\begin{array}{c} A_{R}P_{i^{\prime },R}^{4}+B_{R}P_{i^{\prime },R}^{3}+C_{R}P_{i^{\prime },R}^{2}+D_{R}P_{i^{\prime },R}+E_{R}=0, \end{array}$$

where $A_{R},B_{R},C_{R}$, and $D_{R}$ are coefficients determined by the channel gains and the dual variable $\lambda_{R}$ as

$$\begin{array}{cc} A_{R} & =a_{R},\\ B_{R} & =b_{R}+a_{R}d_{R},\\ C_{R} & =c_{R}\left(a_{R}+1\right)+b_{R}d_{R}-\left(a_{R}d_{R}-b_{R}\right)/\left(2\ln2\lambda_{R}\right),\\ D_{R} & =c_{R}\left(b_{R}+d_{R}\right)-\left(a_{R}-1\right)c_{R}/\left(\ln2\lambda_{R}\right),\\ E_{R} & =c_{R}^{2}-c_{R}\left(b_{R}-d_{R}\right)\left(2\ln2\lambda_{R}\right), \end{array}$$

where $a_{R},b_{R},c_{R}$, and $d_{R}$ are coefficients determined by the channel gains as

$$\begin{array}{cc} a_{R} & =\left(\alpha_{i,A}P_{i,A}+1\right)\left(\alpha_{i,B}P_{i,B}+1\right),\\ b_{R} & =\left(\alpha_{i^{\prime },A}\left(\alpha_{i,B}P_{i,B}+1\right)+\alpha_{i^{\prime },B}\left(\alpha_{i,A}P_{i,A}+1\right)\right)\left(\alpha_{i,A}P_{i,A}+\alpha_{i,B}P_{i,B}+1\right)/\left(\alpha_{i^{\prime },A}\alpha_{i^{\prime },B}\right),\\ c_{R} & ={\left(\alpha_{i,A}P_{i,A}+\alpha_{i,B}P_{i,B}+1\right)}^{2}/\left(\alpha_{i^{\prime },A}\alpha_{i^{\prime },B}\right),\\ d_{R} & =\left(\alpha_{i^{\prime },A}+\alpha_{i^{\prime },B}\right)\left(\alpha_{i,A}P_{i,A}+\alpha_{i,B}P_{i,B}+1\right)/\left(\alpha_{i^{\prime },A}\alpha_{i^{\prime },B}\right). \end{array}$$

The dual variable $\lambda_{R}$ can be obtained by subgradient-based methods with global convergence as the solution of the problem in (23).

### 3.4. Subcarrier Pairing Scheme

Keeping the obtained variables $P_{i,A}^{*},P_{i,B}^{*}$, and $P_{i^{\prime },R}^{*}$, we next determine the subcarrier pairing scheme, which is

(30) $$\begin{array}{cc} \; & \max_{\left\{\rho \right\}}\sum_{i=1}^{N}\sum_{i^{\prime }=1}^{N}\rho_{i,i^{\prime }}R_{i,i^{\prime }}^{S}\\ \; & s.t.\;\sum_{i^{\prime }=1}^{N}\rho_{i,i^{\prime }}=1,\;\forall i,\;\;\sum_{i=1}^{N}\rho_{i,i^{\prime }}=1,\;\forall i^{\prime },, \end{array}$$

which is an integer programming. Defining a N×N cost matrix $R=\left[R_{i,i^{\prime }}^{S}\right],\forall i,i^{\prime }\in \left\{1,...N\right\}$, the solution of problem (30) is finding an optimal assignment of *N* elements in the cost matrix to maximize the cost. The subscript of each selected element in R is corresponding to the subcarrier-pair $\left(i,i^{\prime }\right)$, where the row represents the subcarrier index *i* and the column represents the subcarrier index $i^{\prime }$. Particularly, this selection is a standard linear assignment problem and we can solve it by the Hungarian method [33] with $O(\left(N^{3}\right)$ complexity. We define μ(i) as the optimal subcarrier index in the second slot paired with subcarrier *i* in the first slot, and the optimal subcarrier pairing variable can be given by

(31) $$\rho_{i,i^{\prime }}^{*}=\left\{\begin{array}{c} 1,\;i^{\prime }=\mu \left(i\right)\\ 0,\;\mathrm{otherwise}. \end{array}\right.$$

The μ(i) is obtained by the Hungarian method and (31) is the subcarrier mapping pattern, where the subcarrier mapping scale is N×N. For more details on the Hungarian method, see Appendix D.

### 3.5. Alternating Optimization

**Figure 2:**
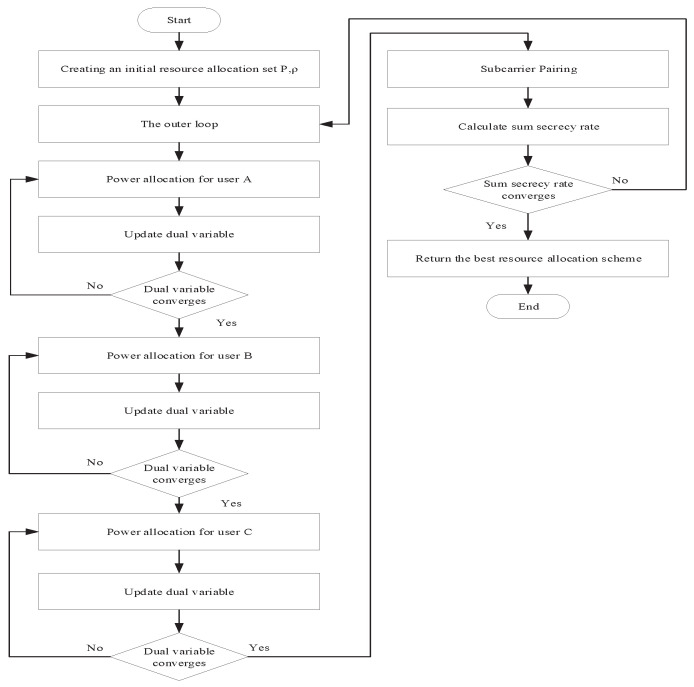
Flowchart of the proposed algorithm.

In the above subsections, the subproblems are solved and the corresponding optimization variables are obtained. Then, we use the AO method to solve the primal optimization problem of joint resource allocation in (16). The initial variable values for the algorithm are $P_{i,A}=P_{A}/N,P_{i,B}=P_{B}/N,P_{i^{\prime },R}=P_{R}/N,i^{\prime }=i,\forall i,i^{\prime }\in \left\{1,...,N\right\}$. Then, in the first loop, we obtain $P_{i,A}^{*}$, $P_{i,B}^{*}$, $P_{i,R}^{*}$ and $\rho_{i,i^{\prime }}^{*}$ by solving the subproblems sequentially. Note that when solving a subproblem, the solution of the previous subproblem is used as its initial variable value. In the next loop, the initial power allocation and subcarrier pairing scheme inherit the results in the previous loop. The loop ends when the iteration count exceeds a threshold. The whole resource allocation algorithm is given in Algorithm 1. The flowchart of the proposed algorithm is shown in Figure 2.

Since the sum secrecy rate of the system increases after each loop and has an upper bound due to the limited power budgets, the sum secrecy rate achieved by the AO algorithm finally converges. Defining the iteration count as γ, the complexity of the AO algorithm is $O(\left(N^{3\gamma }\right)$.
**Algorithm 1** Proposed algorithm for problem (16)1:Initialize $P_{i,A}=P_{A}/N,P_{i,B}=P_{B}/N,P_{i^{\prime },R}=P_{R}/N,i^{\prime }=i,\forall i,i^{\prime }\in \left\{1,...,N\right\}$;2:Initialize $\mu_{A},\mu_{B},\mu_{R}$;3:**for** x=1 to γ **do**4:   **Power Allocation for User** A:5:   **repeat**6:   obtain $P_{i,A}^{*}\left(\mu_{A}\right)$ using (21);7:   update $\mu_{A}$;8:   **until** $\mu_{A}$ converges.9:   **Power Allocation for User** B:10:   **repeat**11:   obtain $P_{i,B}^{*}\left(\mu_{B}\right)$ using (27);12:   update $\mu_{B}$;13:   **until** $\mu_{B}$ converges.14:   **Power Allocation for Relay** R:15:   **repeat**16:   obtain $P_{i^{\prime },R}^{*}\left(\mu_{R}\right)$ using (29);17:   update $\mu_{R}$;18:   **until** $\mu_{R}$ converges.19:   **Subcarrier Pairing:**20:   obtain $\rho^{*}$ according to (31);21:**end for**22:Obtain $\{P^{*}=\left\{P_{i,A}^{*},P_{i,B}^{*},P_{i^{\prime },R}^{*}\right\},\rho^{*}=\left\{\rho_{i,i^{\prime }}^{*}\right\}$.

**Remark** **1.** *There are only two cases for the respective secrecy rate of the user A and B: both 0 or both positive. Particularly, in this optimization algorithm, if $P_{i,A}=0$, then $P_{i,B}=0$, and vice versa. This is because when $P_{i,A}=0$, according to (9), there is no interference to the relay eavesdropping on user A. Therefore, the $R_{i,i^{\prime },A}^{S}=0$, which means that user B does not need to allocate power on the i-th subcarrier, i.e., $P_{i,B}=0$.*

## 4. Simulation Results

In simulation, the signal fading follows the Rayleigh distribution. For simplicity, the power constraints of the users and relay are assumed to be the same. The distance between user A and B is 2 km, and the relay is located at the center of their connection. Three degraded benchmarks, namely, the channel-based power allocation with subcarrier pairing (CBA with SP), equal power allocation with subcarrier pairing (EPA with SP), and equal power allocation without subcarrier pairing (EPA without SP), are considered for comparisons. Since the computational complexity of the algorithms is a polynomial of the number of subcarriers, the sorting scale of the simulation depends on the number of subcarriers, and we perform simulations with subcarriers ranging from 4 to 32.

The CBA with SP algorithm first allocates power according to the equivalent channel coefficient of each subcarrier, with more power allocated to subcarriers with better channels. Then, the algorithm uses the Hungarian method for subcarrier pairing. Differently, the EPA with SP algorithm first allocates power equally on subcarriers and then uses the Hungarian method for subcarrier pairing. The EPA without SP algorithm only allocates power equally on subcarriers, where the subcarrier index for pairing is the same, i.e., $i=i^{\prime }$.

Two conventional algorithms are also presented for comparison. The first algorithm is the SNR-based allocation (SBA) scheme proposed in [7]. This algorithm defines an SNR threshold and assumes that when the eavesdropping SNR of the relay is less than the threshold, the untrusted relay cannot decode the confidential signals, so the eavesdropping rate is 0. Then, the primal non-convex problem can be simplified to a convex problem. The second algorithm is the derivative algorithm (DA) proposed in [17]. This algorithm splits the primal multiple-variable problem into several univariate problems and obtains the solution of these problems by derivation.

In all resource allocation schemes, a central controller acts as service provider, which is assumed to have perfect knowledge of all CSI. The users, as service requesters, provide their own power budgets and CSI to the service provider, and the service provider utilizes this information for subcarrier pairing and power allocation in order to ensure secure communication for users.

**Figure 3:**
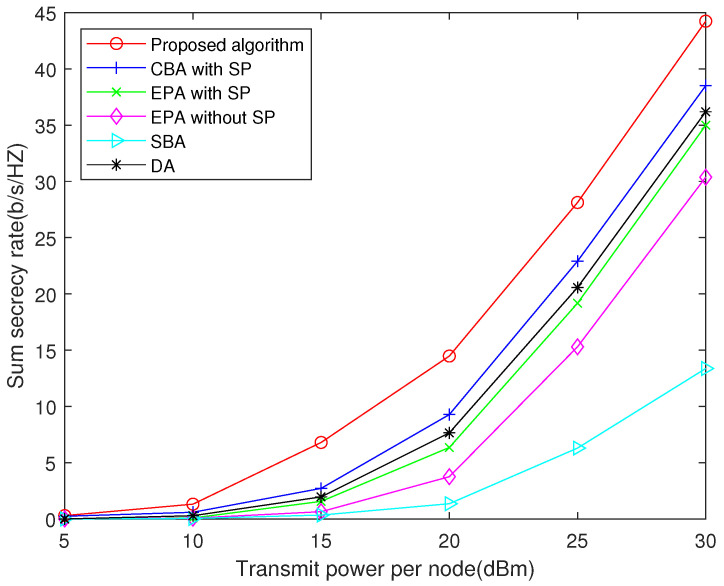
Sum secrecy rate versus transmit power per node when *N* = 16.

The sum secrecy rate achieved by different algorithms is shown in Figure 3. The numerical results are based on average of 200 Monte Carlo simulations. We can see that the proposed algorithm achieves higher sum secrecy rate than the three benchmarks. In a high SNR regime, the proposed algorithm improves the achievable sum secrecy rate of the system by more than 15% over the three degraded benchmarks and two conventional algorithms. Figure 3 also shows that the CBA algorithm performs better than the EPA algorithm, which indicates that the subcarrier pairing can effectively improve secrecy performance of the system. The SBA algorithm has the worst performance, because in the two-way untrusted relay system, the small eavesdropping SNR of the relay will result in the decrease of the transmission rate of the users. Hence, the sum secrecy rate of the system will also be reduced. The performance of the DA algorithm is between the CBA algorithm and the EBA algorithm, because the optimization problem is non-convex and the DA algorithm is only suitable for convex problems.

**Figure 4:**
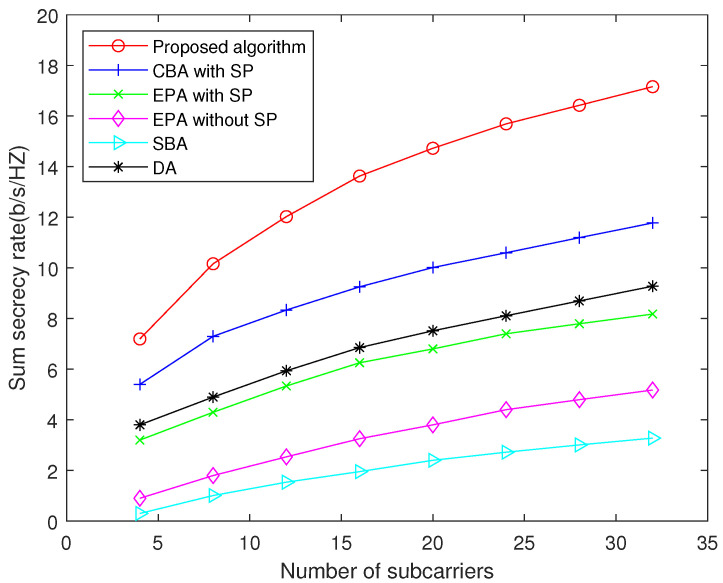
Sum secrecy rate versus number of subcarriers when transmit power per node is 20 dBm.

Figure 4 compares the sum secrecy rate achieved by different algorithms with respect to the number of subcarriers. It can be seen that the proposed algorithm significantly outperforms other schemes, especially with more subcarriers. This is because the conventional algorithms in benchmarks cannot make efficient use of the diversity of subcarriers. Furthermore, since the power and bandwidth of the system are limited, the sum secrecy rate will increase with the growth of the number of subcarriers at first, and finally converge. Therefore, the slope of Figure 4 will gradually decrease.

**Figure 5:**
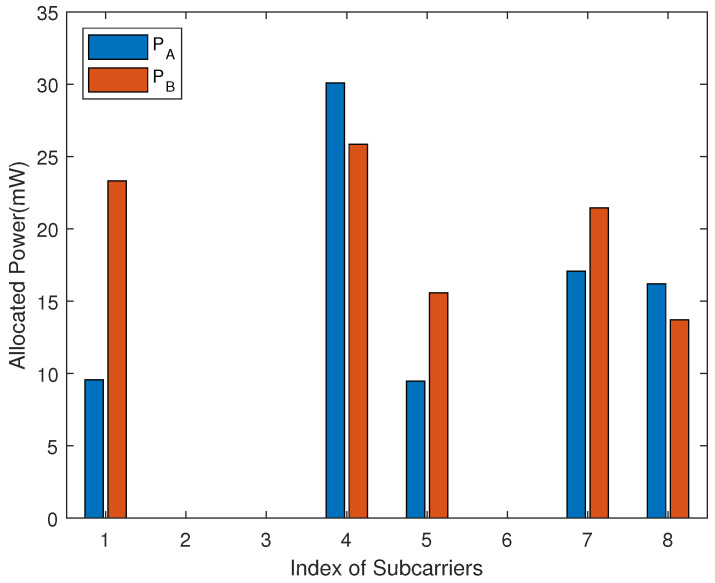
Allocated power versus index of subcarriers when transmit power per node is 20 dBm; *N* = 8.

Figure 5 shows the power allocation scheme on different subcarriers by the proposed algorithm. The corresponding effective channel coefficients of subcarriers are presented in Table 2. It can be seen that for the same subcarrier, if $P_{A}=0$, then $P_{B}=0$, which proves our remark in Section 3. We can also see that the proposed algorithm tends to allocate more power to subcarriers with similar channel gains on A to R and B to R. This is because the untrusted relay decodes one of the users’ signals by treating the other user as noise. Therefore, the allocated power on the subcarrier with similar channel gains on A to R and B to R can more effectively reduce the eavesdropping rate and increase the sum secrecy rate.

**Figure 6:**
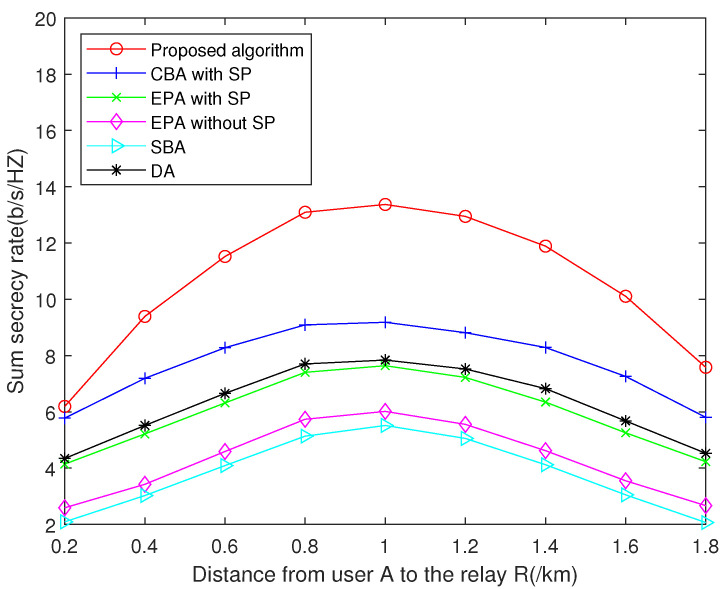
Sum secrecy rate versus distance from user A to the relay R when *N* = 16 and transmit power per node is 20 dBm.

Figure 6 compares the sum secrecy rate achieved by different algorithms with respect to the distance from the user to the relay. The proposed algorithm has better performance than algorithms in the benchmarks at any location of the relay. Particularly, from Figure 6, it can be seen that the sum secrecy rate achieved by all algorithms reaches the maximum when the distance from the relay R to the user A is 1 km, i.e., the distance from A to R is equal to the distance from B to R. This is because when the distances from A to R and B to R are closer, the channel fading parameters of the subcarriers on A to R and B to R become similar. As mentioned above, the allocated power on the subcarrier with similar channel gains on A to R and B to R can more effectively reduce the eavesdropping rate and increase the sum secrecy rate. Therefore, the sum secrecy rate of the system increases as the distances between the relay and the two users become closer.

**Figure 7:**
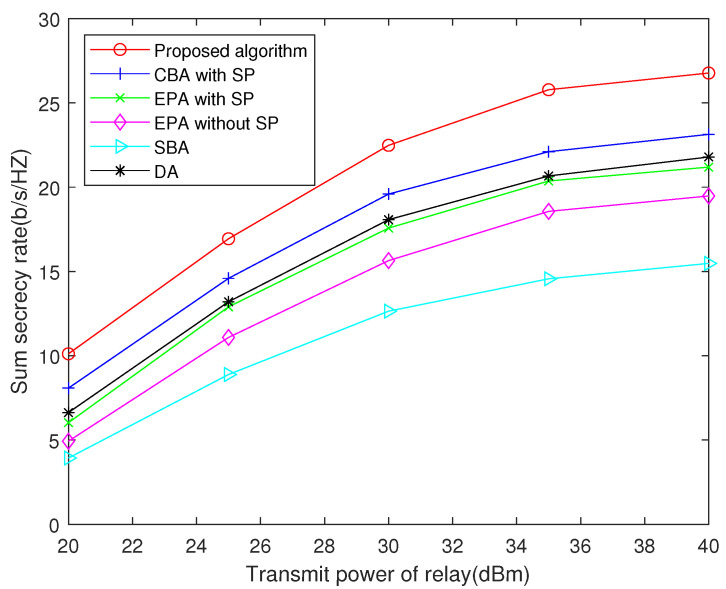
Sum secrecy rate versus transmit power of relay R when *N* = 16 and transmit power of user nodes is 20 dBm.

Particularly, when the untrusted relay is not cooperative and could alter the power to undermine the strategy, the proposed algorithm can still effectively allocate resource, because the power allocation of the untrusted relay is decoupled from the primal problem in the proposed algorithm. Therefore, the proposed algorithm still works well with the variation of the relay power. In Figure 7, we limit the user’s power to 20 dBm, and the untrusted relay can alter its transmit power. Since the untrusted relay is not cooperative, it will always allocate power equally to each subcarrier. Figure 7 compares the sum secrecy rate achieved by different algorithms in this scenario. It can be seen that our proposed algorithm has the best performance, which also confirms our analysis.

## 5. Conclusions

In this paper, we introduced an OFDM two-way untrusted relay system and formulated a subcarrier-pair-based secure resource allocation problem to maximize the sum secrecy rate of the system. A joint resource allocation algorithm based on the AO method was proposed to solve the non-convex optimization problem. We show that the primal NP-hard problem can be solved in polynomial time by decomposing into several subproblems. Furthermore, we show that in this system, there is no situation where one user can achieve secure communication and another user cannot. The simulation results show that the proposed algorithm outperforms other existing algorithms significantly, especially in a high-SNR regime with more subcarriers.

For the future extension, we will consider the smart untrusted relay in the OFDM two-way communication system. This means the relay can use pilot spoofing to alternate channel estimates, masquerading the eavesdropping channel as in [34], so the CSI is imperfect. Effective channel estimators should be used to combat the pilot spoofing attacks.

## Figures and Tables

**Table 1:** A summary of related works.

Reference	Algorithm	Year	Pros and Cons
[7]	SNR-based approach	2016	- Easy implementation
			- Unable to find optimal solution
[16]	Iterative algorithm	2018	- Obtain better solutions
			- High complexity
[17]	Derivative algorithm	2018	- Low complexity
			- Only suitable for univariate problems
[23]	Fractional programming algorithm	2019	- Obtain better solutions
			- Only suitable for fractional problems
[25]	Dual algorithm	2021	- Low complexity
			- Only suitable for convex problems

**Table 2:** Effective channel coefficients of simulation in Figure 5.

*i*	1	2	3	4	5	6	7	8
$\alpha_{i,A}$	2.51	0.80	0.10	1.16	0.72	0.22	1.50	0.45
$\alpha_{i,B}$	0.80	0.27	1.34	1.15	0.39	0.83	0.80	0.59

## Data Availability

The data presented in this study are available on request from the corresponding author. The data are not publicly available due to confidentiality.

## Abbreviations

The following abbreviations are used in this manuscript:

OFDM	Orthogonal frequency division multiple
AF	Amplify and forward
MIMO	Multiple-input–multiple output
AWGN	Additive white Gaussian noise
CSI	Channel state information
SNR	Signal-to-noise ratio
AO	Alternating optimization
CBA	Channel-based power allocation
SP	Subcarrier pairing
EPA	Equal power allocation

## Appendix A

For simplicity, we replace $R_{i,i^{\prime },A}^{S}$ by $R_{a}$, $R_{i,i^{\prime },B}^{S}$ by $R_{b}$, $R_{i,i^{\prime }}^{S}$ by $R_{s}$, $P_{i,A}$ by $P_{a}$, $P_{i^{\prime },R}$ by $P_{r}$, $P_{i,B}$ by $P_{b}$, $\alpha_{i,A}$ by $\alpha_{a}$, $\alpha_{i^{\prime },A}$ by $\alpha_{a^{\prime }}$, $\alpha_{i,B}$ by $\alpha_{b}$, and $\alpha_{i^{\prime },B}$ by $\alpha_{b^{\prime }}$ in the proof, respectively.

(1) When $R_{b}=0$, $R_{b}$ is a concave function of $P_{a}$.

(2) When $R_{b}>0$, it can be rewritten as

(A1) $$\begin{array}{c} R_{b}\left(P_{a}\right)=\frac{1}{2}\log_{2}\left(\frac{a_{a}P_{a}+b_{a}}{P_{a}^{2}+c_{a}P_{a}+b_{a}}\right), \end{array}$$

where

$$\begin{array}{cc} a_{a} & =\left(\alpha_{b^{\prime }}P_{r}+1\right)\left(\alpha_{b}P_{b}+1\right)/\alpha_{a},\\ b_{a} & =\left(\alpha_{b^{\prime }}P_{r}+\alpha_{b}P_{b}+1\right)\left(\alpha_{b}P_{b}+1\right)/\alpha_{a}^{2},\\ c_{a} & =\left(\alpha_{b^{\prime }}P_{r}+2\alpha_{b}P_{b}+2\right)/\alpha_{a}. \end{array}$$

It can be calculated that when $0<P_{a}<P_{a}^{⋄}$, $\frac{\partial R_{b}}{\partial P_{a}}>0$, when $P_{a}>P_{a}^{⋄}$, $\frac{\partial R_{b}}{\partial P_{a}}<0$, where

(A2) $$\begin{array}{c} P_{a}^{⋄}=\frac{-b_{a}\pm \sqrt{a_{a}^{2}b_{a}-a_{a}b_{a}c_{a}+b_{a}^{2}}}{a_{a}}. \end{array}$$

Since our target is to maximize $R_{b}$, the optimal solution $P_{a}^{*}$ will always satisfy $P_{a}^{*}\leq P_{a}^{⋄}$. Hence, the domain of $P_{a}$ can be reduced to $\left[0,P_{a}^{⋄}\right]$. The optimization problem in (18) is equivalent to the problem below, which can be given by

(A3) $$\begin{array}{cc} \; & \max_{\left\{P_{i,A}\right\}}\sum_{i=1}^{N}\left(R_{i,i^{\prime },B}-R_{i,\mathrm{RB}}\right).\\ s.t.\;\sum_{i=1}^{N} & P_{i,A}\leq P_{S},\;P_{i,A}\leq P_{i,A}^{⋄},\forall i. \end{array}$$

The second derivative of $R^{S}\left(P_{s}\right)$ is

(A4) $$\begin{array}{cc} \frac{\partial^{2}R_{b}}{\partial P_{a}^{2}} & =\frac{1}{2\ln2{\left(a_{a}P_{a}+b_{a}\right)}^{2}{\left(P_{a}^{2}+c_{a}P_{a}+b_{a}\right)}^{2}}\\ \; & \cdot \left\{-2{\left(a_{a}P_{a}+b_{a}\right)}^{2}\left(P_{a}^{2}+c_{a}P_{a}+b_{a}\right)\right.\\ \; & \left.+[a_{a}P_{a}^{2}+2b_{a}P_{a}-b_{a}\left(a_{a}-c_{a}\right)]\right.\\ \; & \cdot \left.[3a_{a}P_{a}^{2}+2\left(b_{a}+a_{a}c_{a}\right)P_{a}+b_{a}\left(a_{a}+c_{a}\right)]\right\}. \end{array}$$

When $P_{a}\leq P_{a}^{⋄}$, $a_{a}P_{a}^{2}+2b_{a}P_{a}-b_{a}\left(a_{a}-c_{a}\right)\leq 0$, $\frac{\partial^{2}R_{b}}{\partial P_{a}^{2}}\leq 0$. Thus, we prove $R_{b}$ is a concave function of $P_{a}$ when $P_{a}\in \left[0,P_{a}^{⋄}\right]$. Therefore, the problem in (A3) is convex and the optimization problem in (18) is also convex.

## Appendix B

Substituting $R_{b}$ with $R_{a}$, $P_{a}$ with $P_{b}$, $\alpha_{a}$ with $\alpha_{b}$, $\alpha_{b}$ with $\alpha_{a}$, and $\alpha_{b^{\prime }}$ with $\alpha_{a^{\prime }}$, the proof of Proposition 2 is exactly the same as the proof of Proposition 1.

## Appendix C

(1) When $R_{a}>0$ and $R_{b}>0$, $R_{s}$ can be rewritten as:

(A5) $$\begin{array}{c} R_{s}\left(P_{r}\right)=\underset{R_{s1}}{\underset{︸}{\frac{1}{2}\log_{2}\left(1+\gamma_{a}\right)}}+\underset{R_{s2}}{\underset{︸}{\frac{1}{2}\log_{2}\left(1+\gamma_{b}\right)}}-C, \end{array}$$

where *C* denotes the eavesdropping rate of relay, which is constant when $P_{a}$ and $P_{b}$ is given.

The second derivative of the first part of $R_{s}\left(P_{r}\right)$ is

(A6) $$\begin{array}{c} \frac{\partial^{2}R_{s1}}{\partial P_{r}^{2}}=\frac{1}{2\ln2}\frac{-\left(a_{r}d_{r}-b_{r}c_{r}\right)\left(2a_{r}c_{r}P_{r}+a_{r}d_{r}+b_{r}c_{r}\right)}{{\left(a_{r}P_{r}+b_{r}\right)}^{2}{\left(c_{r}P_{r}+d_{r}\right)}^{2}}, \end{array}$$

where

$$\begin{array}{cc} a_{r} & =\alpha_{b^{\prime }}\left(\alpha_{a}P_{a}+1\right)\left(\alpha_{b}P_{b}+1\right),\\ b_{r} & =\left(\alpha_{a}P_{a}+\alpha_{b}P_{b}+1\right)\left(\alpha_{b}P_{b}+1\right),\\ c_{r} & =\alpha_{b^{\prime }}\left(\alpha_{a}P_{a}+\alpha_{b}P_{b}+1\right),\\ d_{r} & ={\left(\alpha_{a}P_{a}+\alpha_{b}P_{b}+1\right)}^{2}. \end{array}$$

Since $a_{r}d_{r}-b_{r}c_{r}\geq 0$, $\frac{\partial^{2}R_{s1}}{\partial P_{r}^{2}}\leq 0$. Similarly, we can prove $\frac{\partial^{2}R_{s2}}{\partial P_{r}^{2}}\leq 0$. Hence, $\frac{\partial^{2}R_{s}}{\partial P_{r}^{2}}\leq 0$.

(2) When $R_{a}>0$ and $R_{b}=0$, $R_{s}=R_{s1}-C$, $\frac{\partial^{2}R_{s}}{\partial P_{r}^{2}}\leq 0$.

(3) When $R_{a}=0$ and $R_{b}>0$, $R_{s}=R_{s2}-C$, $\frac{\partial^{2}R_{s}}{\partial P_{r}^{2}}\leq 0$.

(4) When $R_{a}=0$ and $R_{b}=0$, $R_{s}=0$, $\frac{\partial^{2}R_{s}}{\partial P_{r}^{2}}=0$.

Therefore, we prove $R_{s}$ is a concave function of $P_{r}$ and the optimization problem in (28) is convex.

## Appendix D. The Hungarian Method

The assignment problem’s goal is to determine the optimum assignment that, e.g., minimizes the total cost. The Hungarian algorithm is an effective algorithm that solves the assignment problem.

In: a N×N matrix, named cost matrix.

Step 1: Subtract row minimum

For each row, find the lowest element and subtract it from each element in that row.

Step 2: Subtract column minimum

Similarly, for each column, find the lowest element and subtract it from each element in that column.

Step 3: Cover all zeros with a minimum number of lines

Cover all zeros in the resulting matrix using a minimum number of horizontal and vertical lines. If *n* lines are required, an optimal assignment exists among the zeros. The algorithm stops.

If less than n lines are required, continue with Step 4.

Step 4: Create additional zeros

Find the smallest element (call it *k*) that is not covered by a line in Step 3. Subtract *k* from all uncovered elements, and add *k* to all elements that are covered twice.
